# Action of Dipeptidyl Peptidase‐4 Inhibitors on SARS‐CoV‐2 Main Protease

**DOI:** 10.1002/cmdc.202000921

**Published:** 2021-02-17

**Authors:** Herbert Nar, Gisela Schnapp, Oliver Hucke, Timothy C. Hardman, Thomas Klein

**Affiliations:** ^1^ Boehringer-Ingelheim Pharma, GmbH & Co. KG Birkendorferstr. 65 88397 Biberach Germany; ^2^ Niche Science & Technology Ltd. 26 Bardolph Road Richmond Surrey UK

**Keywords:** COVID19, DPP-4, drug repurposing, SARS-CoV-2, linagliptin, main protease (M^pro^)

## Abstract

In a recent publication, Eleftheriou et al. proposed that inhibitors of dipeptidyl peptidase‐4 (DPP‐4) are functional inhibitors of the main protease (M^pro^) of SARS‐CoV‐2. Their predictions prompted the authors to suggest linagliptin, a DPP‐4 inhibitor and approved anti‐diabetes drug, as a repurposed drug candidate against the ongoing COVID‐19 pandemic. We used an enzymatic assay measuring the inhibition of M^pro^ catalytic activity in the presence of four different commercially available gliptins (linagliptin, sitagliptin, alogliptin and saxagliptin) and several structural analogues of linagliptin to study the binding of DPP‐4 inhibitors to M^pro^ and their functional activity. We show here that DPP‐4 inhibitors like linagliptin, other gliptins and structural analogues are inactive against M^pro^.

## Graphical abstract

**Getting a second opinion**: In‐silico docking analysis of the main protease (M^pro^) of the SARS‐CoV‐2 structure has suggested that it may be blocked by commercial dipeptidyl peptidase‐4 inhibitors. We used an assay measuring inhibition of M^pro^ catalytic activity in the presence of four different DPP‐4 inhibitors, measuring binding to M^pro^ and functional activity. No activity against M^pro^ was observed.

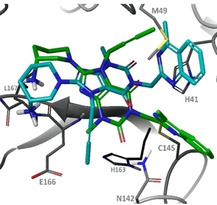

In May, earlier this year, Eleftheriou and colleagues published a manuscript entitled “In Silico Evaluation of the Effectivity of Approved Protease Inhibitors against the Main Protease of the Novel SARS‐CoV‐2 Virus”.^[1]^ Reasoning that the standard process of novel drug development is too lengthy to address the acute medical challenge of a world‐wide pandemic, they proposed in‐silico‐based drug repurposing as an alternative approach. The viral main protease (M^pro^) was selected as target for this purpose, and its 3D structure was compared with that of several candidate human proteases targeted by approved drugs. The authors further reported their docking analysis to the M^pro^ structure of over 30 protease inhibitors that are already approved or under development.

A similarity in 3D structure with M^pro^ was observed for hepatitis C virus protease and alpha‐thrombin. Data from the docking analysis indicated possible activity of inhibitors that target HCV protease, DPP‐4, alpha‐thrombin and coagulation Factor Xa. The authors concluded that, as some of the compounds they investigated are well‐tolerated drugs, their promising in silico results might warrant further evaluation. In particular, Eleftheriou et al. proposed that dipeptidyl peptidase‐4 (DPP‐4) inhibitors with antiviral action might be useful for infected patients with diabetes,^[1]^ a group predominantly susceptible to the disease.

A widely expressed glycoprotein, DPP‐4 acts both as a cell‐membrane‐bound receptor and a soluble enzyme. Being expressed widely, the enzymatic functions of DPP‐4 against a variety of substrates are well‐recognized, including actions on incretin hormones, cytokines, chemokines, neuropeptides and growth factors. In the context of coronavirus infections, membrane‐associated human DPP‐4 has been identified as a functional receptor of middle east respiratory syndrome coronavirus (MERS‐CoV), interacting with MERS‐CoV via the spike glycoprotein S1b domain to promote viral entry.^[2]^ However, for SARS‐CoV‐2 there is strong evidence for angiotensin converting enzyme‐2 as an important functional receptor protein.[^3^, ^4^, ^5^] To our knowledge, such evidence for a similar role of DPP‐4 is lacking.

The M^pro^ active‐site binding mode for linagliptin predicted with our docking workflow deviates substantially from that described by Eleftheriou et al.^[1]^ (Figure S1 in the Supporting Information shows the dependence of the predicted binding geometry on the docking algorithm). This observation fits with our experience that predicted binding modes are not necessarily supported by experimental information (e. g., NMR/X‐ray/SAR data for related chemical matter) and can only serve to propose a hypothesis that needs to be verified experimentally before it can be of any practical use. In addition, using two different 3D similarity search methods we did not identify DPP‐4 as a target related to M^pro^ in terms of their overall three‐dimensional structure and active site topology.

Finally, measurement of inhibition of SARS‐CoV‐2 M^pro^ proteolytic activity by linagliptin, three other gliptins and six closely related analogues of linagliptin (displayed schematically in Figure S2) showed inactivity of all DDP‐4 inhibitors up to the highest tested concentration (500 μM in case of linagliptin, Table S1). The positive control, calpeptin l, was active in the range of 4–5 μM (Figure 1).

**Figure 1:**
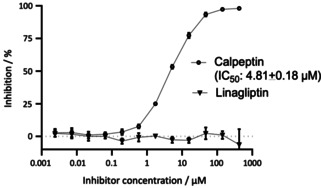
Inhibition curve of SARS‐CoV‐2 M^pro^ with calpeptin or linagliptin. Data shown are mean (±SD) values from three independent experiments.

In summary, we show here that the tested DPP‐4 inhibitors like linagliptin, three other gliptins and six structural linagliptin analogues are inactive against M^pro^. As discussed above, this outcome does not surprise us, especially as there is no apparent structural similarity between the two target proteins. Our de‐validation of DPP‐4 inhibitors as SARS‐CoV‐2 M^pro^ inhibitors serves to underline the limitations of ligand docking in terms of identifying candidate compounds when undertaking drug‐repurposing projects, it must be viewed as only one potential first step. Although experimental validation of the predictions remains critical, our findings do not, for example, preclude any observed activity of gliptins against SARS‐CoV‐2, which might be a consequence of other actions.^[6]^

## Conflict of interest

H.N., O.H., G.S. and T.K. are employees of Boehringer‐Ingelheim Pharma, the manufacturer of linagliptin but otherwise the authors declare no conflict of interest.

## Supporting information

As a service to our authors and readers, this journal provides supporting information supplied by the authors. Such materials are peer reviewed and may be re‐organized for online delivery, but are not copy‐edited or typeset. Technical support issues arising from supporting information (other than missing files) should be addressed to the authors.

Supplementary

## References

[1] P. Eleftheriou , D. Amanatidou , A. Petrou , A. Geronikaki , Molecules 2020, 25, 2529. 10.3390/molecules25112529 PMC7321236 32485894

[2] V. S. Raj , H. Mou , S. L. Smits , H. W. Dekkers , M. A. Müller , R. Dijkmman , D. Muth , J. A. A. Demmer , A. Zaki , R. A. M. Fouchier , Nature 2013, 495, 251–254. 23486063 10.1038/nature12005 PMC7095326

[3] M. Hoffmann , H. Kleine-Weber , S. Schroeder , N. Krüger , T. Herrler , S. Erichsen , T. S. Schiergens , G. Herrler , N−H. Wu , A. Nitsche , Cell 2020, 181, 271–280. 32142651 10.1016/j.cell.2020.02.052 PMC7102627

[4] R. Yan , Y. Zhang , Y. Li , L. Xia , Y. Guo , Q. Zhou , Science. 2020, 367, 1444–1448. 32132184 10.1126/science.abb2762 PMC7164635

[5] C. Lei , K. Qian , T. Li , S. Zhang , W. Fu , M. Ding , S. Hu , Nat. Commun. 2020, 11, 2070. 32332765 10.1038/s41467-020-16048-4 PMC7265355

[6] S. B. Solerte , F. D'Addio , R. Trevisan , E. Lovati , A. Rossi , I. Pastore , M. Dell'Acqua , E. Ippolito , C. Scaranna , R. Bellante , Diabetes Care 2020, 43, 2999–3006. 32994187 10.2337/dc20-1521 PMC7770266

