# Supplementary material for: Action of Dipeptidyl Peptidase‐4 Inhibitors on SARS‐CoV‐2 Main Protease
Source: ChemMedChem. 2021 Feb 17;16(9):1425–6. doi: 10.1002/cmdc.202000921 (PMC8248156; doi:10.1002/cmdc.202000921)
Supplement: Supplementary file 1 — Supplementary [file CMDC-16-1425-s001.pdf]

ChemMedChem

Supporting Information

Action of Dipeptidyl Peptidase-4 Inhibitors on SARS-CoV-2 Main Protease

Herbert Nar, Gisela Schnapp, Oliver Hucke, Timothy C. Hardman, and Thomas Klein*

Supporting Information

Materials and Methods

Docking of linagliptin to Mpro

We used publicly available coordinate of the Mpro structure (PDB-code 6m2n) that were also used by Eleftheriou et al. [1]. Hydrogen atoms were added using the protein preparation module in Maestro (Schrödinger, LLC, New York, NY Release 2020-2). The docking program Glide (Schrödinger, LLC) was used in SP mode to predict 10 potential binding modes for linagliptin [2–4]. The highest ranked pose based on Glide Score was selected as the predicted binding mode. The binding model proposed by Eleftheriou et al. was reproduced on basis of their description [1]: linagliptin was placed manually in the active site of Mpro, followed by an optimization of the binding geometry with the "Refine protein-ligand complex" module of Maestro.

3D similarity searches for targets related to Mpro

GESAMT (General Efficient Structural Alignment of Macromolecular Targets), employing the QScore scoring function [5], and CavBase [6, 7] were used to search for targets related to Mpro including DPP-4.

Biochemical Assay testing Mpro activity

The SARS-CoV-2 Mpro (3264-3569aa) protein at a final concentration of 20 nM (Proteros, Munich, Germany) was incubated with 10 µL reaction buffer (20 mM HEPES pH 7.5, 1 mM DTT, 1 mM EDTA, 100 mM NaCl, 0.01% Tween20) in 384 well plates (Corning, 4514). Aliquots of 85 nL of DPP-4 inhibitor DMSO stock solutions (Boehringer Ingelheim, Germany; Carbosynth Ltd, UK) were added to the wells and incubated for 30 min. Reactions were initiated by addition of Dabcyl-KTSAVLQSGFRKM-E(Edans)-NH2 substrate (final assay concentration 5 µM). After 60 min incubation at RT, the increase of fluorescence intensity was detected. Values for the 50% inhibition concentration (IC_50) were calculated from concentration response curves determined over a 1 nM to 100 µM, or to 500 µM in the case in for Linagliptin range. Data were analyzed with the GraphPad Prism version 8 software (San Diego, CA 92108, USA).

Supplementary Table 1: Structures and inhibitory activities of the DPP-4 inhibitors investigated in this study.

Compound		IC_{50} (μM)	IC_{50} error (μM)
Calpeptin		4,81	0,182
Sitagliptin	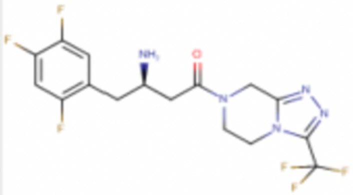	>100	NA
Alogliptin	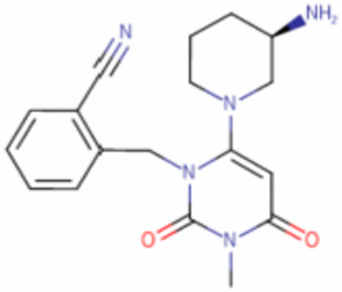	>100	NA
Saxagliptin	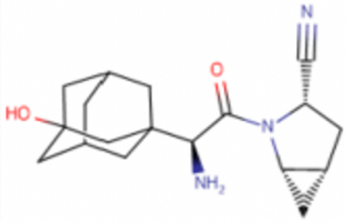	>100	NA
Linagliptin	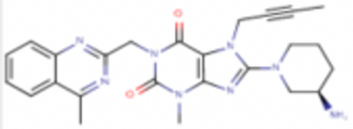	>500	NA

Supplementary Figure 1: Two predicted binding modes of linagliptin in SARS-CoV-2 Mpro. Cyan: Model based on Eleftheriou et al [1].; Green: Prediction with Glide in this work. In both cases the X-ray structure 6m2n.pdb was used as receptor structure.

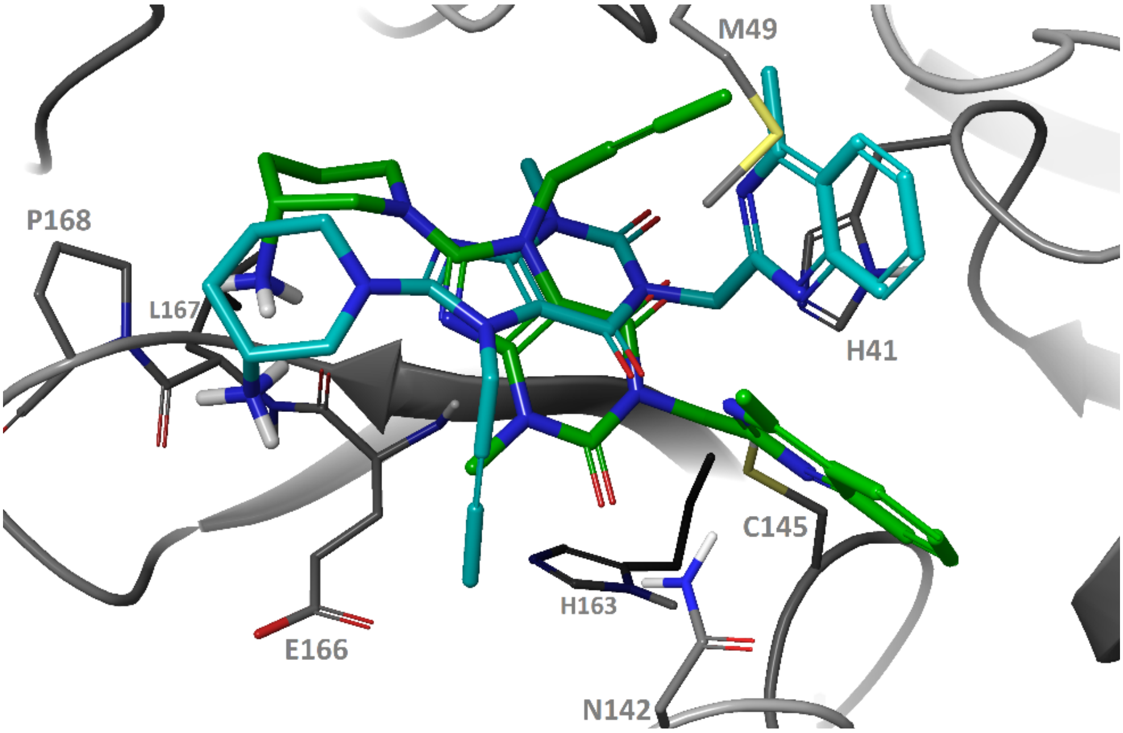

Supplementary Figure S2: Scheme of variation points in the linagliptin structure that were explored in various analogues

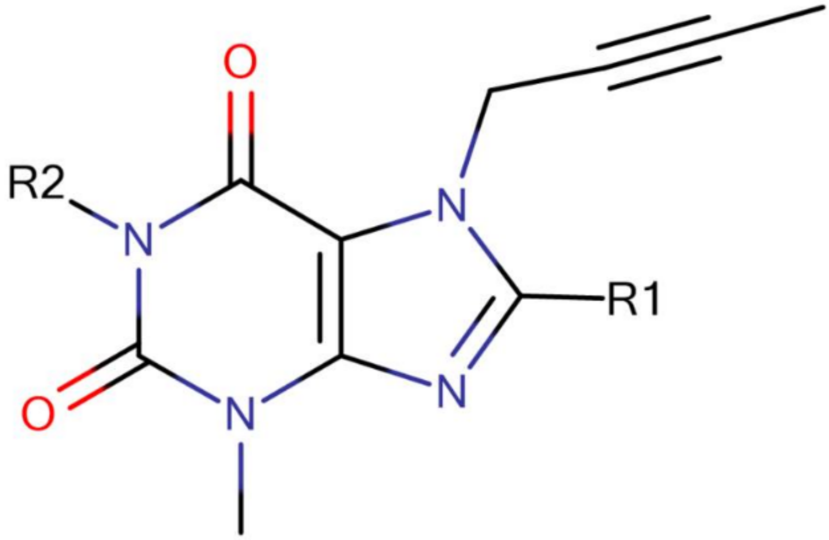

References

1. Eleftheriou, P.; Amanatidou, D.; Petrou, A; Geronikaki, A. In Silico Evaluation of the Effectivity of Approved Protease Inhibitors against the Main Protease of the Novel SARS-CoV-2 Virus. Molecules. 2020, 25, 2529; doi:10.3390/molecules25112529.
2. Friesner, R. A.; Banks, J. L.; Murphy, R. B.; Halgren, T. A.; Klicic, J. J.; Mainz, D. T.; Repasky, M. P.; Knoll, E. H.; Shaw, D. E.; Shelley, M.; Perry, J. K.; Francis, P.; Shenkin, P. S. Glide: A New Approach for Rapid, Accurate Docking and Scoring. 1. Method and Assessment of Docking Accuracy, J. Med. Chem., 2004, 47, 1739–1749. doi: 10.1021/jm0306430.
3. Halgren, T. A.; Murphy, R. B.; Friesner, R. A.; Beard, H. S.; Frye, L. L.; Pollard, W. T.; Banks, J. L. Glide: A New Approach for Rapid, Accurate Docking and Scoring. 2. Enrichment Factors in Database Screening. J. Med. Chem. 2004, 47, 1750–1759. doi: 10.1021/jm030644s.
4. Friesner, R. A.; Murphy, R. B.; Repasky, M. P.; Frye, L. L.; Greenwood, J. R.; Halgren,T. A.; Sanschagrin, P. C.; Mainz, D. T. Extra Precision Glide: Docking and Scoring Incorporating a Model of Hydrophobic Enclosure for Protein-Ligand Complexes. J. Med. Chem. 2006, 49, 6177–6196. doi.org/10.1021/jm051256o.
5. Krissinel, E. Enhanced fold recognition using efficient short fragment clustering. J. Mol. Biochem. 2012, 1, 76–85.
6. Schmitt, S .; Kuhn, D.; Klebe, G. A new method to detect related function among proteins independent of sequence and fold homology. J. Mol. Biol. 2002, 323, 387–406. doi: 10.1016/s0022-2836(02)00811-2.
7. Kuhn, D.; Weskamp, N.; Schmitt, S.; Hullermeier, E; Klebe, G. From the Similarity Analysis of Protein Cavities to the Functional Classification of Protein Families Using Cavbase. J. Mol. Biol. 2006, 359, 1023–1044. doi: 10.1016/j.jmb.2006.04.024.
